# Preschool children’s asthma medication: parental knowledge, attitudes, practices, and adherence

**DOI:** 10.3389/fphar.2024.1292308

**Published:** 2024-04-03

**Authors:** Jianlan Tang, Zhihua Zhao, Rong Guo, Chao Niu, Renfei Zhang, Ling Wang, Nan Luo

**Affiliations:** Department of Respiratory, Children’s Hospital of Chongqing Medical University, National Clinical Research Center for Child Health and Disorders, Ministry of Education Key Laboratory of Child Development and Disorders, Chongqing Engineering Research Center of Stem Cell Therapy, Chongqing, China

**Keywords:** knowledge, attitudes, practice, asthma, children, treatment adherence and compliance, cross-sectional study

## Abstract

**Introduction:** As parents or legal guardians primarily care for children with asthma, understanding their knowledge, attitudes, and practices (KAP) barriers to treatment and medication adherence is of essential importance. This study aimed to analyze the KAP toward asthma medication and adherence among preschool-aged asthmatic children’s parents and explore the factors influencing adherence.

**Methods:** This cross-sectional study was conducted between February 2023 and April 2023. Parents of preschool children with asthma were asked to complete the questionnaire containing knowledge, attitude, practice dimensions, and demographic characteristics. The Morisky Medication Adherence Scale (MMAS) was used to investigate adherence.

**Results:** A total of 632 valid questionnaires (154 male and 478 female) were included. Parents showed moderate knowledge (9.49 ± 2.86, 63.27%, possible range: 0-15) and moderate attitudes (26.18 ± 2.51, 74.80%, possible range: 7-35) towards asthma medication, while their practices (27.46 ± 5.26, 91.53%, possible range: 6-30) were proactive; however, medication adherence was low (4.84 ± 1.78, total score: 8). The attitude scores (OR = 1.10, 95% CI: 1.01-1.19, P=0.020), practice scores (OR = 1.16, 95%CI: 1.12-1.21, *p* < 0.001), and smoking (OR = 1.64, 95%CI: 1.14-2.37, *p* = 0.008) were associated with medication adherence.

**Discussion:** Preschool-aged asthmatic children’s parents showed moderate knowledge, attitudes, and proactive practice toward asthma medication. Continuous training and education programs should be provided for parents to improve asthma medication management in preschool children.

## Introduction

Asthma is a long-term condition characterized by coughing, wheezing, shortness of breath, and chest tightness, varying in intensity and frequency. These symptoms are accompanied by chronic airway inflammation and variable airflow obstruction (National Asthma and Prevention, 2007; Healthcare Improvement Scotland, 2019; Global Initiative for Asthma, 2023). According to recent statistics, more than 300 million people worldwide are affected by asthma (Papi et al., 2018), and the incidence has been increasing in children and adolescents (Papi et al., 2018; Zahran et al., 2018). In the United States, the prevalence rate of asthma has been over 8% in the last two decades (Zahran et al., 2018), while in China, the prevalence in children increased from 0.91% in 1990 to 3.02% in 2010 (National Collaborative Group on Pediatric Asthma, 2004; Asthma, 2013). Viral respiratory infections, allergens, and tobacco smoke are common triggers for asthma attacks (National Asthma and Prevention, 2007; Healthcare Improvement Scotland, 2019; Global Initiative for Asthma, 2023). Although asthma-related mortality is generally low, patients with multiple psychosocial risk factors and severe asthma may have a higher mortality risk.

The pharmacological management of asthma involves a stepwise approach to therapy, in which pharmacotherapy is adjusted as necessary, and inhaled short-acting beta-2 agonists (SABAs) are prescribed as reliever medication to all children with symptomatic asthma unless they are on maintenance and reliever therapy with inhaled corticosteroids (ICS)-formoterol (National Asthma and Prevention, 2007; Healthcare Improvement Scotland, 2019; Global Initiative for Asthma, 2023). The current Global Asthma Management and Prevention Strategy (GINA) guidelines also suggest anti-inflammatory reliever therapy for asthma exacerbations (Global Initiative for Asthma, 2023). Treatment adherence is crucial for achieving optimal respiratory outcomes, preventing attacks, and reducing healthcare costs (Pearce and Fleming, 2018; Kaplan and Price, 2020; Wong and Mortin, 2021).

Preschoolers represent a special group of patients with asthma. Indeed, accurately assessing symptoms in younger children is difficult because children lack clarity when asked to describe their feelings. In addition, the immature respiratory system can influence the detection of signs and symptoms. Also, lung function tests cannot be performed, and several childhood diseases (e.g., bronchiolitis) can complicate the diagnosis. Therefore, diagnostic methods require the objective documentation of signs or convincing reports from the parents without suspicion of an alternate diagnosis (Cave and Atkinson, 2014; Ducharme et al., 2015; Yang et al., 2021). Furthermore, patients developing asthma at a very young age are at high risk of decreased respiratory function and lung diseases (early loss of lung function) in adulthood; thus, accurate asthma management is crucial for prognosis (Montuschi and Barnes, 2011; Belgrave et al., 2014; Trivedi and Denton, 2019).

The knowledge, attitudes, and practices (KAP) methodology can help identify and assess the barriers and misconceptions of a particular population regarding a specific subject (World Health Organization, 2008; Andrade et al., 2020). It provides quantitative and qualitative data that can be used to design and implement effective training or educational interventions. Previous studies have shown that although parents’ or legal guardians’ KAP regarding childhood asthma was moderate, their understanding of asthma medication was inadequate (Zhao et al., 2002; Ho et al., 2003; Zhao et al., 2013; AlOtaibi and AlAteeq, 2018; Noureddin et al., 2019; Alhammad et al., 2020; Alruwaili et al., 2021). Moreover, poor KAP regarding asthma medication was linked to inadequate asthma control in children (Ho et al., 2003; Alhammad et al., 2020) and poor medication compliance (Zhao et al., 2002). As parents or legal guardians primarily care for children with asthma, understanding their KAP barriers to treatment and medication adherence is of essential importance.

This study aimed to analyze the KAP toward asthma medication and adherence among preschool-aged asthmatic children’s parents and explore the factors influencing adherence.

## Materials and methods

### Participants

This study was conducted between February 2023 and April 2023 at our Hospital (respiratory department). Parents of preschool children with asthma were asked to complete the questionnaire containing knowledge, attitude, practice dimensions, and demographic characteristics. The study was advertised in the waiting rooms. The parents could approach the investigator or the research team if interested. The Ethics Committee approved the study [approval # (2023) Lunshen (Research) No. (Lajunen et al., 2019)]. Written informed consent was obtained from the study subjects before completing the survey. All eligible and willing-to-participate participants during the time frame were enrolled.

The inclusion criteria were the following: (Global Initiative for Asthma, 2023): parents of preschool children (1–6 years old) diagnosed with asthma according to the GINA (Global Initiative for Asthma, 2021; Healthcare Improvement Scotland, 2019) voluntarily participated in this study; (Papi et al., 2018); proficient in using WeChat to answer the questionnaire. The patients with serious physical or mental illnesses prevented from cooperating were excluded. The diagnostic criteria in young children are 1) symptom patterns including wheezing, cough, breathlessness (seen as activity limitation), nocturnal symptoms or awakenings, 2) risk factors for asthma including a family history of atopy, allergic sensitization, allergy or asthma, or a personal history of food allergy or atopic dermatitis, and 3) exclusion of alternate diagnoses (Global Initiative for Asthma, 2021) (the 2021 version was the current one when the study was designed).

### Questionnaire design

The questionnaire was designed by the authors for the purpose of the present study with reference to previous literature exploring parental attitudes toward asthma and factors related to children’s medication adherence (van Dellen et al., 2008; Ponieman et al., 2009; Klok et al., 2015; Koste et al., 2015). A random sample of 66 parents was tested for reliability; Cronbach’s α was 0.805, suggesting high internal consistency.

The final questionnaire was in Chinese, and it contained four dimensions: demographic characteristics (age, gender, ethnicity, education, work type, residence, marital status, monthly *per capita* income, whether anyone in the household smoking, family history of asthma, the severity and duration of asthma, and asthma medication) and knowledge, attitude, and practice dimensions (Supplementary Material). The knowledge dimension consisted of 15 questions: 0 points for wrong or unclear answers, 1 point for correct answers, and a total score ranging from 0 to 15 points. The attitude dimension had seven questions: positive attitudes (items A1, A2, A4, A5, and A6) were normally scored with scores ranging from 5–1, indicating a strong agreement to strong disagreement, and negative attitudes (items A3 and A7) were scored reversely with scores ranging from 1 to 5 indicating a strong agreement to strong disagreement; the total score range of the attitude dimension was 7–35. The practice dimension contained 14 items, and items P1-P6 were scored using a five-point Likert scale, with always to never being assigned a score of 5 to 1, with a score range of 6–30. The Morisky Medication Adherence Scale (MMAS) (Morisky et al., 2008) was used to investigate adherence (Yan et al., 2014). The maximum score was 8, where <6 was considered poor adherence, a score between 6 and 7 was considered moderate adherence, and a score of 8 was considered good adherence.

### Questionnaire distribution and quality control

The Sojump online platform was used, and the questionnaire was verified after data were imported into the platform. The questionnaires were distributed to the study participants by scanning the QR code onsite via WeChat. A specific IP address could be used to submit a questionnaire only once. Instructions and clarifications were given on the requirements for completing the questionnaire before it was distributed, and any doubts about the questions were answered by trained staff at any time during the questionnaire process.

The questionnaires were automatically anonymized and given serial numbers. Answers to all items were mandatory for submission. The questionnaires with obvious filling patterns (e.g., all first choices), logical errors, and those that took <2 min to complete were excluded.

### Statistical analysis

Statistical analysis was performed using STATA 17.0 (Stata Corporation, College Station, TX, United States). The continuous variables were first tested for normality using the Kolmogorov-Smirnov test; the continuous variables conforming to the normal distribution were analyzed using ANOVA (three or more groups) and Student’s t-test (two groups) and presented as means ± standard deviation. Those variables not conforming to the normal distribution were analyzed by Kruskal–Wallis analysis of variance (three or more groups) or Wilcoxon-Mann-Whitney *U*-test (two groups) and presented as medians (ranges). The categorical variables were presented as n (%) and analyzed using Fisher’s exact or Chi-square tests. Pearson correlation was used to analyze the correlation among the KAP dimensions. Logistic multivariable regression was performed with medication adherence as the dependent variable to analyze the factors associated with medication adherence. Medication adherence was divided into ≥6 points (moderate to good adherence) and <6 (poor adherence), after which a forest plot was made. Two-sided *p*-values <0.05 were considered statistically significant.

## Results

### General characteristics

Among a total of 640 collected questionnaires, 8 were excluded as the parents of those children were <18 years old, resulting in 632 valid questionnaires. Most participants were <35 years of age (64.87%), female (75.63%), Chinese Han (93.35%), employed with stable jobs (55.22%), living in urban areas (84.97%), married (98.42%), with a junior college/bachelor’s degree (62.03%), with a monthly income of 5,000–10,000 CNY (45.73%), smoking (62.18%), and without a family history of asthma (68.51%). Most of their children had a history of asthma of 1–3 years (48.73%), had intermittent asthma (48.26%), and were prescribed one drug for asthma (84.65%) (Table 1). The most common drugs were fluticasone propionate (inhalation powder) (62.34%) and fluticasone propionate inhaled aerosol and salmeterol xinafoate (26.42%) (Table 2).

**TABLE 1:** Characteristics of the participants.

	N (%)	Knowledge		Attitude		Practice		Medication adherence	
		Score	*p*	Score	*p*	Score	*p*	Score	*p*
All	632	9.49 ± 2.86		26.18 ± 2.51		27.46 ± 5.26		4.84 ± 1.78	
Parent’s age			0.425		0.396		0.174		0.868
<35	410 (64.87)	9.47 ± 2.73		26.14 ± 2.52		27.26 ± 5.31		4.84 ± 1.82	
≥35	222 (35.13)	9.51 ± 3.10		26.26 ± 2.50		27.83 ± 5.15		4.85 ± 1.73	
Parent’s gender			0.036		0.254		0.032		0.637
Male	154 (24.37)	9.05 ± 3.02		25.86 ± 2.41		28.25 ± 5.22		4.88 ± 1.84	
Female	478 (75.63)	9.63 ± 2.80		26.29 ± 2.54		27.20 ± 5.25		4.83 ± 1.77	
Ethnicity			0.263		0.568		0.912		0.865
Han	590 (93.35)	9.53 ± 2.80		26.18 ± 2.50		27.48 ± 5.31		4.85 ± 1.78	
Minority	42 (6.65)	8.83 ± 3.68		26.19 ± 2.70		27.22 ± 5.25		4.76 ± 1.91	
Work type			<0.001		0.007		0.478		0.764
Employed stable job	349 (55.22)	10.02 ± 2.68		26.46 ± 2.48		27.33 ± 5.41		4.89 ± 1.71	
Unemployed job	283 (44.78)	8.83 ± 2.96		25.84 ± 2.52		27.62 ± 5.07		4.79 ± 1.87	
Residence			0.008		0.035		0.744		0.988
Urban	537 (84.97)	9.63 ± 2.77		26.29 ± 2.43		27.47 ± 5.21		4.85 ± 1.78	
Rural	95 (15.03)	8.66 ± 3.22		25.56 ± 2.84		27.37 ± 5.54		4.82 ± 1.83	
Marital Status			0.699		0.340		0.948		0.334
Married	622 (98.42)	9.49 ± 2.87		26.19 ± 2.50		27.47 ± 5.23		4.84 ± 1.79	
Divorced	8 (1.27)	9.38 ± 2.20		25.25 ± 2.71		26.50 ± 6.85		4.75 ± 1.67	
Widowed	2 (0.32)	8.00 ± 4.24		27.50 ± 4.95		27.75 ± 9.90		6.50 ± 0.71	
Education level			<0.001		<0.001		0.753		0.766
High school and below	208 (32.91)	8.40 ± 2.88		25.54 ± 2.45		27.58 ± 5.34		4.75 ± 1.91	
Junior college/bachelor’s degree	392 (62.03)	10.07 ± 2.60		26.58 ± 2.43		27.40 ± 5.29		4.88 ± 1.72	
Master’s degree or above	32 (5.06)	9.34 ± 3.75		25.47 ± 2.92		27.42 ± 4.48		5.03 ± 1.71	
Monthly *per capita* income, CNY			0.012		0.011		0.165		0.931
<5,000	171 (27.06)	8.89 ± 2.98		25.68 ± 2.27		27.52 ± 5.27		4.82 ± 1.74	
5,000–10,000	289 (45.73)	9.75 ± 2.64		26.30 ± 2.58		7.09 ± 5.34		4.85 ± 1.81	
10,000–20,000	124 (19.62)	9.57 ± 2.83		26.31 ± 2.46		27.75 ± 4.97		4.78 ± 1.87	
>20,000	48 (7.59)	9.81 ± 3.56		26.92 ± 2.80		28.70 ± 5.33		5.02 ± 1.62	
Smoking			0.605		0.428		0.600		0.042
Yes	393 (62.18)	9.43 ± 2.89		26.22 ± 2.51		27.32 ± 5.43		4.94 ± 1.79	
No	239 (37.82)	9.57 ± 2.82		26.13 ± 2.51		27.68 ± 4.96		4.69 ± 1.76	
Family history of asthma			0.016		0.704		0.137		0.308
Yes	87 (13.77)	9.92 ± 2.83		26.46 ± 2.76		27.92 ± 5.58		5.09 ± 1.78	
No	433 (68.51)	9.56 ± 2.89		26.15 ± 2.49		27.60 ± 5.17		4.80 ± 1.77	
Unclear	112 (17.72)	8.88 ± 2.73		26.12 ± 2.40		26.54 ± 5.30		4.82 ± 1.84	
Duration of asthma in children			0.003		0.110		0.160		0.135
<6 months	57 (9.02)	9.18 ± 3.02		25.93 ± 2.34		27.62 ± 4.56		4.53 ± 1.89	
6 months - 1 year	218 (34.49)	9.00 ± 2.94		26.11 ± 2.36		28.04 ± 5.19		4.64 ± 1.94	
1–3 years	308 (48.73)	9.87 ± 2.72		26.15 ± 2.64		27.17 ± 5.41		5.02 ± 1.65	
>3 years	49 (7.75)	9.57 ± 2.97		27.00 ± 2.42		26.52 ± 5.22		4.98 ± 1.66	
The severity of asthma in children			0.147		0.092		0.081		0.681
Intermittent	305 (48.26)	9.70 ± 2.92		26.21 ± 2.49		28.02 ± 5.13		4.72 ± 1.91	
Mildly persistent	260 (41.14)	9.30 ± 2.79		26.30 ± 2.57		27.10 ± 5.36		4.97 ± 1.66	
Moderately persistent	64 (10.13)	9.36 ± 2.82		25.53 ± 2.29		26.29 ± 5.30		4.91 ± 1.67	
Severe persistent	3 (0.47)	7.00 ± 3.00		28.00 ± 1.00		27.00 ± 3.03		5.33 ± 0.58	
Prescribed asthma medications (n)			0.844		0.350		0.301		0.109
1	535 (84.65)	9.48 ± 2.91		26.22 ± 2.48		27.46 ± 5.31		4.89 ± 1.77	
2	81 (12.82)	9.44 ± 2.66		25.93 ± 2.70		27.09 ± 5.08		4.47 ± 1.87	
3	16 (2.53)	9.81 ± 2.34		26.31 ± 2.47		29.36 ± 4.08		5.25 ± 1.53	

**TABLE 2:** Medication status of children with asthma (multiple choice, number).

Medications	N = 632
Salmeterol xinafoate and fluticasone propionate powder for inhalation (fixed dose)	167
Budesonide and formoterol fumarate powder for inhalation (fixed dose)	71
Fluticasone propionate inhaled pMDI	394
Salbutamol sulfate pMDI	63
*Dermatophagoides farinae* drops	11
Other	39
None	0

pMDI: pressurized metered dose asthma inhaler.

### KAP

The knowledge score was 9.49 ± 2.86 (63.27%, possible score range: 0–15), indicating moderate knowledge. Higher knowledge scores were observed in females, participants who were employed and with a stable job, living in urban areas, with a higher education level and higher income, with a family history of asthma, and with longer child’s asthma history (all *p* < 0.05) (Table 1). As shown in Supplementary Table S1, the knowledge items with the lowest rates of correct answers were K8 (19.30%, “When a child is exposed to asthma triggers, he or she should wait before taking the medication until symptoms appear”), K12 (32.92%, “Oral medication works as quickly as inhaled medication”), K10 (37.97%, “Long-term inhalation of glucocorticoids is the most effective way to prevent asthma attacks in children”), K13 (40.66, “Inhaled medication has fewer side effects than oral medication”), K11 (42.25%, “Inhaled glucocorticosteroids should be used even when your child is not having an asthma attack”), K3 (54.59%, “3 or more repeated wheezing episodes indicate asthma”), and K2 (59.49%, “Asthma is a neurological or psychological disorder”).

The attitude score was 26.18 ± 2.51 (74.80%, possible score range: 7–35), indicating moderate attitudes. Higher attitude scores were observed in participants with employed stable jobs, junior college/bachelor’s degrees, living in urban areas, and higher income (all *p* < 0.05) (Table 1). The practice score was 27.46 ± 5.26 (91.53%, possible score range: 6–30), indicating proactive practice. Higher practice scores were also observed in males (*p* = 0.032) (Table 1).

The knowledge scores were correlated to practice and attitude scores (practice scores: r = 0.145, *p* < 0.001; attitude scores: r = 0.359, *p* < 0.001). The attitude scores were correlated to the practice scores (r = 0.251, *p* < 0.001) (Table 3).

**TABLE 3:** Correlation analysis between KAP dimensions.

	Knowledge	Attitude	Practice
Knowledge	1		
Attitude	0.359 (*p* < 0.001)	1	
Practice	0.145 (*p* < 0.001)	0.251 (*p* < 0.001)	1

### Factors associated with children’s medication adherence

**FIGURE 1:**
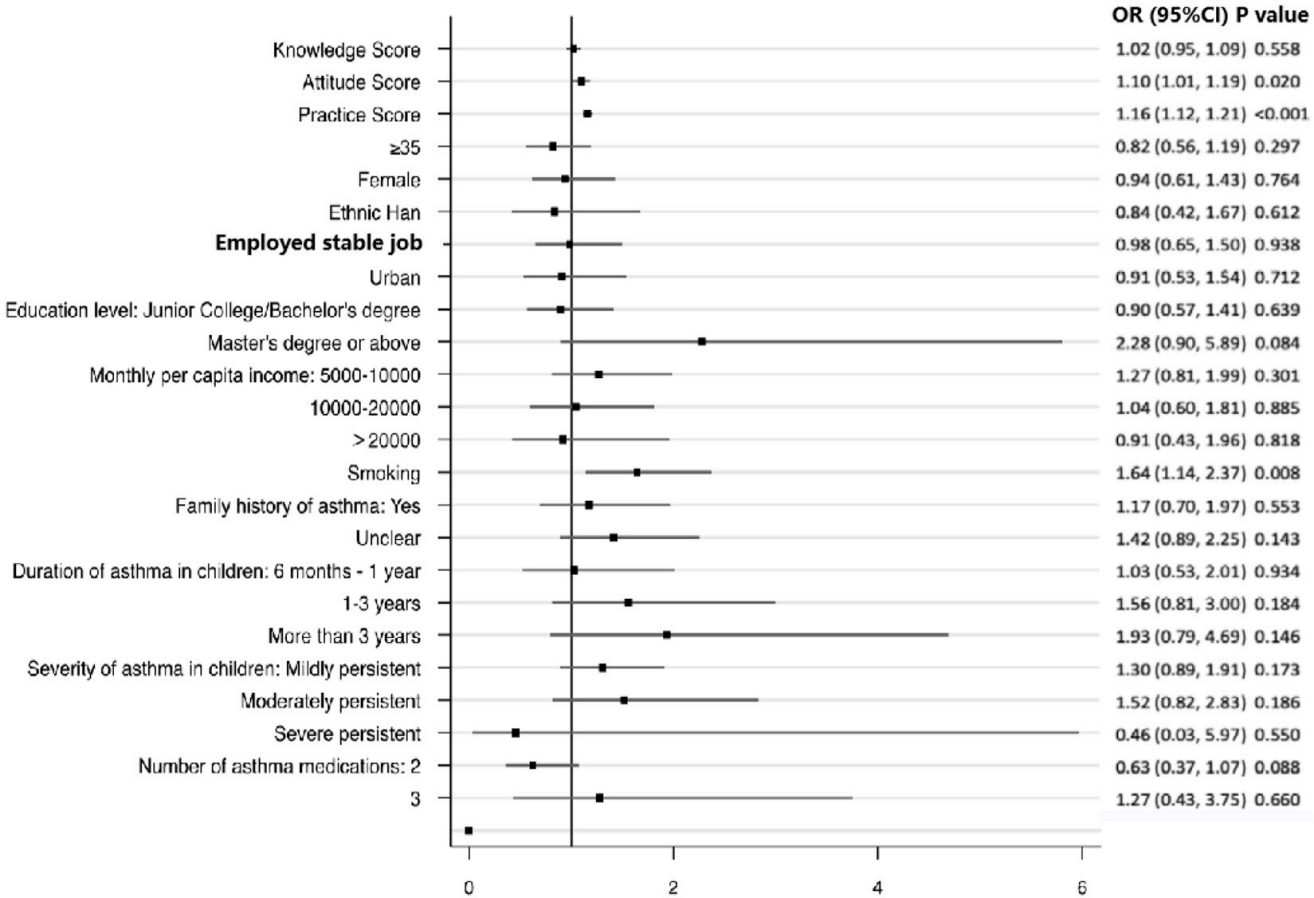
Multivariable analysis of medication adherence. References: <35 years old, female, minority, non-fixed job, rural, high school or below, <5000 CNY, no smoking, no family history of asthma, duration of asthma in children <6 months, severity of asthma in children (intermittent), and a number of asthma medications (Global Initiative for Asthma, 2023).

As shown in Figure 1, the attitude scores (OR = 1.10, 95%CI: 1.01–1.19, *p* = 0.020), practice scores (OR = 1.16, 95%CI: 1.12–1.21, *p* < 0.001), and smoking (OR = 1.64, 95%CI: 1.14–2.37, *p* = 0.008) were associated with medication adherence.

## Discussion

Our data showed that the parents of preschoolers in the Southwest region of Chongqing (China) with asthma showed moderate knowledge, attitudes, and active practice toward asthma medication. The attitude and practice scores, as well as smoking, were associated with treatment adherence.

Most participants were <35 years old, which is consistent with the requirement for their children to be <6 years old, as indicated by a previous study (Al-Binali et al., 2010). Also, previous studies showed that a family history of asthma is a crucial risk factor for developing the condition (Hijazi et al., 2000); however, most participants in this study reported no such history. Additionally, the socioeconomic status of the participants was relatively high.

Previous analysis has shown that parents of children with asthma generally have poor KAP toward asthma medication. For instance, a study conducted in Saudi Arabia found that while knowledge about asthma was moderate, knowledge about asthma medication was poor, with several misconceptions prevailing (AlOtaibi and AlAteeq, 2018; Alhammad et al., 2020). Similarly, a Khartoum (Sudan) study found that mothers admitted to asthma or emergency departments had inadequate knowledge about asthma (Noureddin et al., 2019). Surprisingly, even in developed countries like the United States, a lack of knowledge about asthma and its treatments has been reported (Ho et al., 2003). A systematic review of eight studies also revealed that poor parental KAP on asthma care was prevalent in both Eastern and Western countries, where being a mother, of young age, and with low socioeconomic status were associated with inadequate KAP (Alruwaili et al., 2021). In Nanjing (China), 55% of parents had poor knowledge of asthma and its management (Zhao et al., 2002), while 29 studies across China showed similar results (Zhao et al., 2013). In contrast to previous studies, we found that parents in the Southwest region of Chongqing had moderate knowledge, attitudes, and active practices toward asthma medication. This could be attributed to the relatively higher socioeconomic status of the participants (DeWalt et al., 2007; Gong et al., 2014) and the fact that most of the children in the study were <6 years old and had a history of asthma for 1–3 years. Previous studies presented above-included preschoolers but were not focused on preschoolers, which could influence the results. Nevertheless, the study identified some areas that require education among parents of children with asthma, such as the nature of asthma, triggers, and treatments. The women had higher knowledge scores, while the men had higher practice scores. There are gender differences in access to healthcare, use of healthcare, and help-seeking behaviors between genders (Mauvais-Jarvis et al., 2020). The difference in the knowledge score is consistent with the literature showing a higher health literacy in women compared with men (Clouston et al., 2017; Lee et al., 2015; K et al., 2019). A study showed that men were using more healthcare services than women (Cameron et al., 2010; Azad et al., 2020). Still, the present study was not designed to examine the parents’ gender differences in the management of childhood asthma, but it will be worth studying in the future.

Treatment adherence is crucial for children with asthma (Pearce and Fleming, 2018; Kaplan and Price, 2020; Wong and Mortin, 2021), particularly in preschoolers (Belgrave et al., 2014; Yang et al., 2021). To the best of our knowledge, there are no reports on data assessing the relationship between parental KAP and asthma control, specifically in preschoolers. A study conducted in Saudi Arabia by Alhammad et al. (2020) reported a correlation between poor asthma control and poor parental KAP. However, Ho et al. (2003) reported no association between KAP and asthma outcomes or treatment adherence in United States patients in general. In China, Zhao et al. (2002) found a correlation between poor KAP and poor medication compliance in children. Many parents are concerned about the long-term effects of corticosteroids on their children, including side effects, impacts on development, and possible dependence (Orrell-Valente et al., 2007; Zhao et al., 2013).

In the present study, higher practice and attitude scores, as well as smoking, were associated with greater adherence among parents to asthma medication for their preschoolers. As previously reported, the association between attitude and practice scores and adherence is not surprising (Adams, 2010; Carrillo Zuniga et al., 2012). Yet, the association with smoking is somewhat surprising. Some studies suggested that exposure to secondary smoke is a significant risk factor for asthma development in children (National Asthma and Prevention, 2007; Healthcare Improvement Scotland, 2019; Global Initiative for Asthma, 2023) and has a negative effect on asthma control (Soussan et al., 2003; Gerald et al., 2009). Tobacco exposure can affect lung function in preschoolers with asthma for as long as 10 years after exposure (Lajunen et al., 2019). Nonetheless, parents may be aware of the harmful effects of smoking on preschool children, prompting them to adhere more to treatment to prevent asthma attacks or make them smoke outdoors to protect their children. Ensuring high adherence may be easier for them than quitting smoking.

The present study has a few limitations. First, only a small proportion of Chinese children were enrolled (Xiaoyan and Shuxia, 2020). Also, only participants from the Southwest region of Chongqing, China, were enrolled. Only the number of prescribed drugs was collected, not their formulation nor their frequency of use. Furthermore, our results reflect a single point in time (World Health Organization, 2008; Andrade et al., 2020), but they can serve as a baseline to examine the impact of future educational interventions. As with any KAP survey, there is a possibility of social desirability bias, in which participants may provide socially acceptable answers instead of their actual behaviors (Bergen and Labonte, 2020). Finally, lung function data of children were not collected, which could have provided valuable insights into the impact of treatment adherence on asthma control.

Parents of preschool asthmatic children from the Southwest region of Chongqing, China, showed moderate knowledge, attitudes, and proactive practices toward asthma medication. The attitude and practice scores and smoking were associated with treatment adherence. It is recommended that continuous training and education programs be provided for parents to improve the management of asthma medication among preschool children.

## Scope statement

This study aimed to investigate the knowledge, attitude, and practice of parents of preschool children with asthma towards their children’s medication adherence and to explore the factors associated with medication adherence. A cross-sectional study was conducted between February 2023 and April 2023 at the respiratory department of the Children’s Hospital Affiliated to Chongqing Medical University, China. A final questionnaire was developed, containing four dimensions: demographic characteristics, knowledge dimension, attitude dimension, and practice dimension. The Morisky Medication Adherence Scale (MMAS) was used to investigate adherence. The study enrolled 632 parents of preschool children with asthma, and the majority of the participants were female, Chinese Han, living in urban areas, with a junior college/bachelor’s degree, and a monthly income of 5,000–10,000. The results showed that the knowledge and practice scores were low, while the attitude scores were relatively high. Furthermore, logistic multivariable regression showed that low monthly *per capita* income, poor knowledge, negative attitude, and poor practice were independent risk factors for poor adherence to asthma medication. The findings suggest that interventions should be implemented to improve parental knowledge, attitude, and practice toward asthma medication adherence, particularly among those with low monthly *per capita* income.

## Data Availability

The original contributions presented in the study are included in the article/Supplementary Material, further inquiries can be directed to the corresponding author.
