# Supplementary material for: Preschool children’s asthma medication: parental knowledge, attitudes, practices, and adherence
Source: Front Pharmacol. 2024 Apr 3;15:1292308. doi: 10.3389/fphar.2024.1292308 (PMC11021651; doi:10.3389/fphar.2024.1292308)
Supplement: Supplementary file 1 [file Table1.DOCX]

**Supplementary Table S1.** Correct rate of knowledge.

|  | n (%) |
| --- | --- |
| 1. Asthma is a chronic inflammatory disease that requires long-term treatment | 548 (86.71) |
| 1. Asthma is a neurological or psychological disorder | 376 (59.49) |
| 1. 3 or more repeated wheezing episodes indicate asthma | 348 (55.06) |
| 1. Asthma in children often occurs without warning | 345 (54.59) |
| 1. Diet and environment can also cause asthma in children | 512 (81.01) |
| 1. Passive smoking in children can cause or worsen asthma attacks | 515 (81.49) |
| 1. Asthma attacks will be less frequent if the child’s asthma triggers are identified and avoided | 572 (90.51) |
| 1. When a child is exposed to asthma triggers, he or she should wait before taking medication until symptoms appear | 122 (19.30) |
| 1. Although asthma cannot be cured, it can be controlled with the right medication | 540 (85.44) |
| 1. Long-term inhalation of glucocorticoids is the most effective way to prevent asthma attacks in children | 240 (37.97) |
| 1. Inhaled glucocorticosteroids should be used even when your child is not having an asthma attack | 267 (42.25) |
| 1. Oral medication works as quickly as inhaled medication | 227 (35.92) |
| 1. Inhaled medication has fewer side effects than oral medication | 257 (40.66) |
| 1. If your child does not have an asthma attack, there is no need for regular follow-up visits to the clinic | 547 (86.55) |
| 1. Regular lung function tests can monitor changes in your child’s condition | 579 (91.61) |
