# Supplementary material for: Preschool children’s asthma medication: parental knowledge, attitudes, practices, and adherence
Source: Front Pharmacol. 2024 Apr 3;15:1292308. doi: 10.3389/fphar.2024.1292308 (PMC11021651; doi:10.3389/fphar.2024.1292308)
Supplement: Supplementary file 2 [file Table2.DOC]

**Preschool Children’s Asthma Medication: Parental Knowledge, Attitudes, Practices, and Adherence**

**Basic Information**

| 1. Parent’s age, years |
| --- |
| 1. <35 |
| b. ≥35 |
| 2. Parent’s gender |
| a. Male |
| b. Female |
| 3. Ethnicity |
| a. Han |
| b. Minority |
| 4. Work type |
| a. Employed stable job |
| b. Unemployed job |
| 5. Residence |
| a. Urban |
| b. Rural  6. Marital status |
| a. Married |
| b. Divorced |
| c. Widowed |
| 7. Education level |
| a. High school and below |
| b. Junior college/bachelor’s degree |
| c. Master’s degree or above |
| 8. Monthly per capita income, CNY |
| a. <5000 |
| b. 5000-10,000 |
| c. 10,000-20,000 |
| d. >20,000 |
| 9. Smoking |
| a. Yes |
| b. No |
| 10. Family history of asthma |
| a. Yes |
| b. No |
| c. Unclear |
| 11. Duration of asthma in children |
| a. <6 months |
| b. 6 months - 1 year |
| c. 1-3 years |
| d. >3 years |
| 12. The severity of asthma in children |
| a. Intermittent |
| b. Mildly persistent |
| c. Moderately persistent |
| d. Severe persistent |
| 13. Prescribed aAsthma medications |
| a. 1 |
| b. 2 |
| c. 3 |
| 14. Medication status of children with asthma (multiple choicer) |
| a. Salmeterol xinafoate and fluticasone propionate powder for inhalation (fixed dose) |
| b. Budesonide and formoterol fumarate powder for inhalation (fixed dose) |
| c. Fluticasone propionate inhaled pMDI |
| d. Salbutamol sulfate pMDI |
| e. *Dermatophagoides farinae* drops |
| f. Other |
| g. None |

**Knowledge Assessment**

| 1. Asthma is a chronic inflammatory disease that requires long-term treatment | a.True | b.False | c.Unclear |
| --- | --- | --- | --- |
| 2. Asthma is a neurological or psychological disorder | a.True | b.False | c.Unclear |
| 3. 3 or more repeated wheezing episodes indicate asthma | a.True | b.False | c.Unclear |
| 4. Asthma in children often occurs without warning | a.True | b.False | c.Unclear |
| 5. Diet and environment can also cause asthma in children | a.True | b.False | c.Unclear |
| 6. Passive smoking in children can cause or worsen asthma attacks | a.True | b.False | c.Unclear |
| 7. Asthma attacks will be less frequent if the child’s asthma triggers are identified and avoided | a.True | b.False | c.Unclear |
| 8. When a child is exposed to asthma triggers, he or she should wait before taking medication until symptoms appear | a.True | b.False | c.Unclear |
| 9. Although asthma cannot be cured, it can be controlled with the right medication | a.True | b.False | c.Unclear |
| 10. Long-term inhalation of glucocorticoids is the most effective way to prevent asthma attacks in children | a.True | b.False | c.Unclear |
| 11. Inhaled glucocorticosteroids should be used even when your child is not having an asthma attack | a.True | b.False | c.Unclear |
| 12. Oral medication works as quickly as inhaled medication | a.True | b.False | c.Unclear |
| 13. Inhaled medication has fewer side effects than oral medication | a.True | b.False | c.Unclear |
| 14. If your child does not have an asthma attack, there is no need for regular follow-up visits to the clinic | a.True | b.False | c.Unclear |
| 15. Regular lung function tests can monitor changes in your child’s condition | a.True | b.False | c.Unclear |

**Attitude Assessment**

| 1. I believe it is essential to have an understanding of asthma. | a. Strongly agree | b. Agree | c. Neutral | d. Disagree | e. Strongly disagree |
| --- | --- | --- | --- | --- | --- |
| 2. I believe it is necessary to acquire knowledge about asthma medications, such as how to operate inhalation devices. | a. Strongly agree | b. Agree | c. Neutral | d. Disagree | e. Strongly disagree |
| 3. I think long-term medication has a negative impact on a child's growth. | a. Strongly agree | b. Agree | c. Neutral | d. Disagree | e. Strongly disagree |
| 4. I believe improving the child's living environment is necessary, such as quitting smoking. | a. Strongly agree | b. Agree | c. Neutral | d. Disagree | e. Strongly disagree |
| 5. I believe inhaled medications are as effective as expected. | a. Strongly agree | b. Agree | c. Neutral | d. Disagree | e. Strongly disagree |
| 6. I believe the child's emotional and mental well-being has improved after medication. | a. Strongly agree | b. Agree | c. Neutral | d. Disagree | e. Strongly disagree |
| 7. I am concerned that the child may develop a dependence on asthma medications. | a. Strongly agree | b. Agree | c. Neutral | d. Disagree | e. Strongly disagree |

**Practice Assessment**

| 1. Before the child inhales medication, I will clean the child's mouth and wash their face. | a.Always | b.Often | c. Sometimes | d. Occasionally | e.Never |
| --- | --- | --- | --- | --- | --- |
| 2. During the child's inhalation of medication, I will instruct the child on the correct breathing technique and observe if it is being done accurately. | a.Always | b.Often | c. Sometimes | d. Occasionally | e.Never |
| 3. While administering inhaled medication, I will monitor the child's complexion and breathing. | a.Always | b.Often | c. Sometimes | d. Occasionally | e.Never |
| 4. After inhaling medication, I will ensure the child rinses their mouth promptly, perform oral care, and clean the child's face. | a.Always | b.Often | c. Sometimes | d. Occasionally | e.Never |
| 5. Following inhalation medication, I will facilitate effective coughing and phlegm expulsion in the child, such as turning them over and patting their back. | a.Always | b.Often | c. Sometimes | d. Occasionally | e.Never |
| 6. After inhalation medication, I will promptly clean the nebulizer device and let it air-dry for future use. | a.Always | b.Often | c. Sometimes | d. Occasionally | e.Never |
| Pediatric Medication Adherence Survey | | | | | |
| 7. Does your child frequently forget to take medication? | a. Yes | b. No |  |  |  |
| 8. In the past 2 weeks, has there been a day or several days when your child forgot to take medication? | a. Yes | b. No |  |  |  |
| 9. If you notice that your child's condition worsens after taking medication, have you ever reduced or stopped the medication without informing the doctor? | a. Yes | b. No |  |  |  |
| 10. When your child travels or is away from home for an extended period, do they sometimes forget to carry their treatment medications and devices? | a. Yes | b. No |  |  |  |
| 11. Did your child undergo treatment yesterday? | a. Yes | b. No |  |  |  |
| 12. When you believe your child's condition is under control, do you allow them to stop taking medication? | a. Yes | b. No |  |  |  |
| 13. Daily treatment can be very inconvenient for some people. Does your child dislike undergoing treatment every day? | a. Yes | b. No |  |  |  |
| 14. How frequently does your child forget to undergo asthma medication treatment? | a. Always | b. Often | c. Sometimes | d. Very rarely | e. No |
